# A transcriptomic signature that predicts prehypertension in adolescence and higher systolic blood pressure in childhood

**DOI:** 10.1172/jci.insight.192837

**Published:** 2025-12-08

**Authors:** Reena Perchard, Terence Garner, Philip G. Murray, Amirul Roslan, Lucy E. Higgins, Edward D. Johnstone, Adam Stevens, Peter E. Clayton

**Affiliations:** 1 Division of Developmental Biology & Medicine, Faculty of Biology, Medicine & Health, University of Manchester, Manchester, United Kingdom.; 2 Manchester University NHS Foundation Trust, Manchester, United Kingdom.

**Keywords:** Cardiology, Public Health, Hypertension, Preventative medicine

## Abstract

**BACKGROUND:**

Suboptimal fetal growth (SFG), being born small for gestational age (SGA), and catch-up (CU) growth are, individually and together, linked to cardiometabolic risks. However, not all develop adverse outcomes. This study aimed to validate a transcriptomic signature to identify individuals at greatest cardiometabolic risk.

**METHODS:**

Using National Heart, Lung and Blood Institute (NHLBI) criteria to define cardiometabolic risk, healthy and prehypertensive 17-year-olds were identified in the Avon Longitudinal Study of Parents and Children (ALSPAC) (UK) childhood cohort. Epigenomic and transcriptomic differences were analyzed. A hypergraph identified functionally related genes, which were used in random forest classification to predict prehypertensive phenotypes. The BabyGRO (UK) cohort included 80 children aged 3–7 years, born at term following pregnancies with SFG risks. Anthropometric and cardiometabolic markers and transcriptomic profiles were collected, fetal and childhood weight trajectories and their relationship to cardiometabolic markers were assessed, and transcriptome was used for prediction.

**RESULTS:**

Individuals with CU-SGA in ALSPAC were 1.6 times more likely than all others to be prehypertensive at 17 years (*P* < 1 × 10^–5^). A 42-gene hypergraph cluster was highly predictive of prehypertension (AUC 0.984, error rate 5.4%). In BabyGRO, 20 of these genes accurately predicted higher systolic blood pressure (AUC 0.971, error rate 3.6%). This transcriptomic signature could help identify children with adverse pre- and postnatal growth who may develop prehypertension.

**CONCLUSION:**

A blood transcriptomic signature exists in childhood which distinguishes those at risk of adult cardiometabolic disease among children with adverse pre- and postnatal growth.

**TRIAL REGISTRATION:**

Regional ethics committee reference 17/NW/0153, IRAS project ID 187679.

**FUNDING:**

Centre grant to the Maternal and Fetal Health Research Centre by Tommy’s The Pregnancy and Baby Charity, Child Growth Foundation, European Research Council funding as part of the Health and Environment-wide Associations based on Large Population Surveys (HEALS) study

## Abstract 2

We have discovered genes connecting poor growth in the womb to higher blood pressure in children and teenagers, helping detect heart health risks earlier.

## Graphical abstract

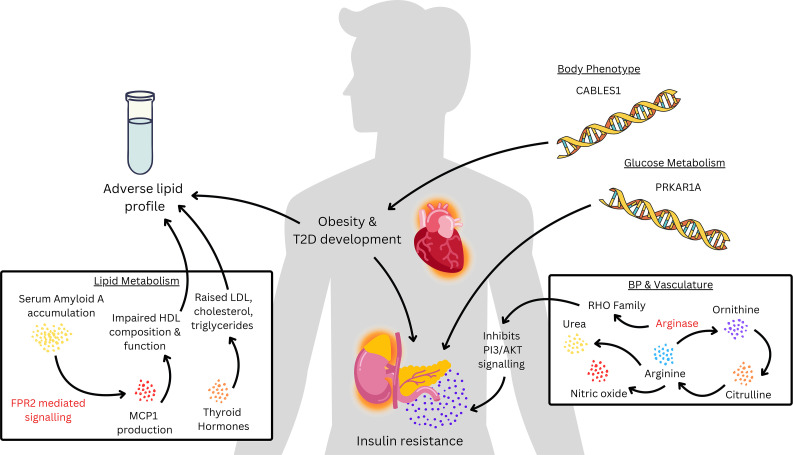

## Introduction

Metabolic adaptations that help a fetus survive its adverse intrauterine environment can persist into postnatal life. In the presence of plentiful resources, these confer a disadvantage to that individual. First proposed as the fetal origins hypothesis by Barker et al. (1), this became known as the Developmental Origins of Health and Disease (DOHaD), a field encompassing studies of epigenetic, transcriptomic, metabolomic, and phenotypic consequences of events that occurred from conception onward (2). Many studies that build on and provide further support for the Barker hypothesis have focused on recruiting individuals who are small for gestational age (SGA), others have recruited large cohorts of healthy participants (3, 4). Individuals with birthweights at the lower end of this range have provided valuable insights, demonstrating changes in glucose metabolism, lipid metabolism, and vascular health markers.

Across these studies SGA, defined in a number of ways — for example, less than the tenth percentile, third percentile, or a SD score less than –2 — is accepted as a marker of fetal growth restriction (FGR). However, a fetus can undergo suboptimal fetal growth (SFG) — i.e., fail to reach its genetically determined growth potential — without being born small. Furthermore, accelerated postnatal growth, both with and without SGA (5), in the presence or absence of prenatal undernutrition (6) is recognized as disadvantageous, emphasising a particular limitation of studies recruiting only those born SGA. Thus, there is a need to include those individuals who have experienced SFG and not necessarily born small in studies defining relationships between fetal, postnatal growth, and cardiometabolic risk.

Animal studies have played a role in identifying epigenetic clues of functional links between ‘omics and outcomes, with transcription factors such as *PPARA* (7), *PDX1* (8), *HNF4A* (9), and *NR3C* (10) highlighted. Transcriptomic studies in animals have also supported the relevance of *HNF* transcription factors (11), and highlighted genes related to the regulation of blood pressure and arachidonic acid metabolism (12). In humans, a number of isolated epigenetic (13) and transcriptomic studies (14) have been performed, in attempts to reveal complex biological pathways.

**Figure 1:**
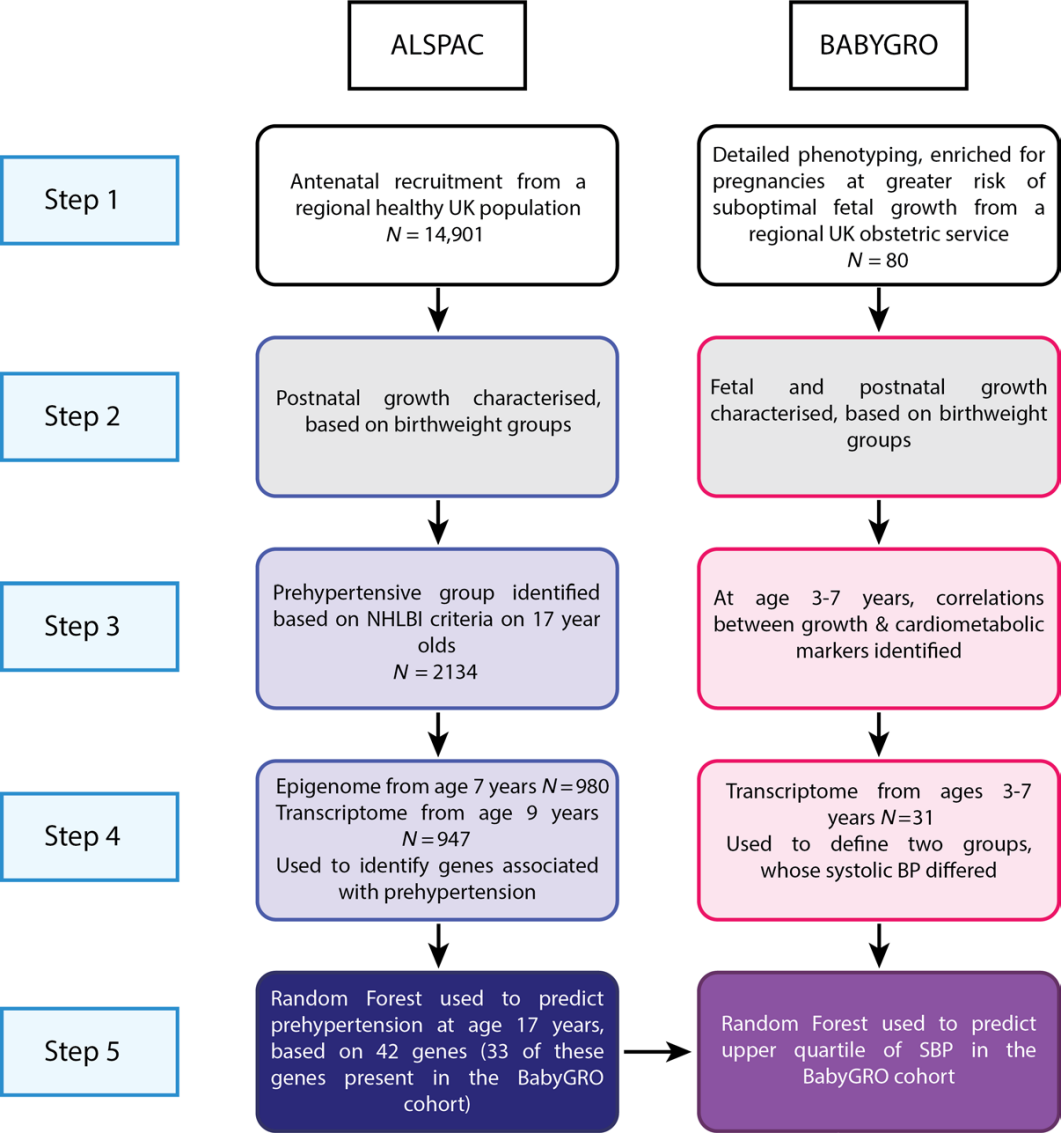
Summary of methods used for ‘omic analyses. ALSPAC. Step 1. Antenatal recruitment and an additional attempt to boost recruitment when the oldest children were aged 7 years resulted in a total sample size of 14,901 who were alive at 1 year, where birthweight data were available. Step 2. Postnatal growth was characterized. Step 3. NHLBI criteria were used to define the prehypertensive group at age 17 years. Step 4. Based on the epigenome (age 7 years) and transcriptome (age 9 years), a hypergraph refined a central cluster of genes, which were tested using a random forest model. Step 5. 42 genes were found to be predictive of prehypertension in ALSPAC. BabyGRO. Step 1. Pregnant women were streamlined into antenatal research clinics, resulting in availability of detailed ultrasound data, including estimated fetal weight. Step 2. Fetal and postnatal growth was characterized. Step 3. Correlations were established between growth trajectories and cardiometabolic markers in childhood. Step 4. Based on transcriptome data from children, 2 groups were defined and systolic blood pressure (SBP) was found to differ between these groups, justifying the use SBP as the outcome variable for a random forest model. Step 5. Genes predictive of a prehypertensive phenotype in ALSPAC were tested for their ability to predict higher SBP in BabyGRO.

To expand our understanding of the growth trajectories and underlying mechanisms and molecular pathways that underpin DOHaD, we are using 2 populations of healthy children; the first has been derived from the Avon Longitudinal Study of Parents and Children (ALSPAC), focusing on those born SGA who show catch-up (CU) growth over childhood, on whom longitudinal growth and health data are available through childhood and adolescence. The second population includes (a) babies born to mothers whose early pregnancy fetoplacental hormone profiles indicate a risk of SFG and (b) babies born following normal pregnancies, whose early postnatal growth has been monitored (the BabyGRO cohort; Figure 1 and Table 1).

Our aims were (a) to define the later-life cardiometabolic risk in the ALSPAC SGA cohort; (b) to use their transcriptomic signature in midchildhood to predict those with increased cardiometabolic risk in the late teenage years; (c) to characterize fetal and postnatal growth trajectories, cardiometabolic risk, and transcriptomic profiles in an early life cohort enriched for SFG; and (d) to use this cohort to test and validate the ALSPAC prediction model.

## Results

**Figure 2:**
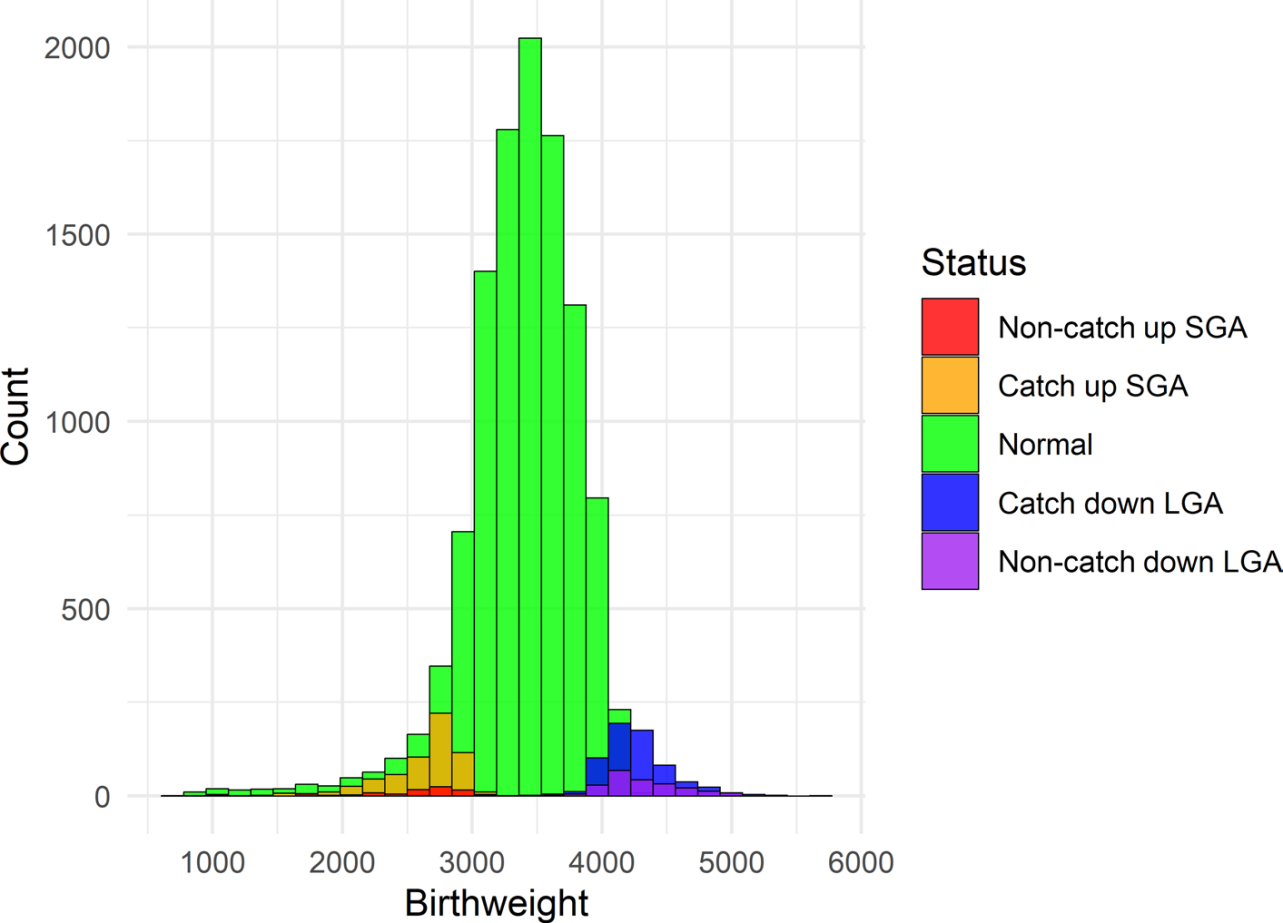
Birthweight histogram. The distribution of birthweights among the children who participated in the Avon Longitudinal Study of Parents and Children study.

**Figure 3:**
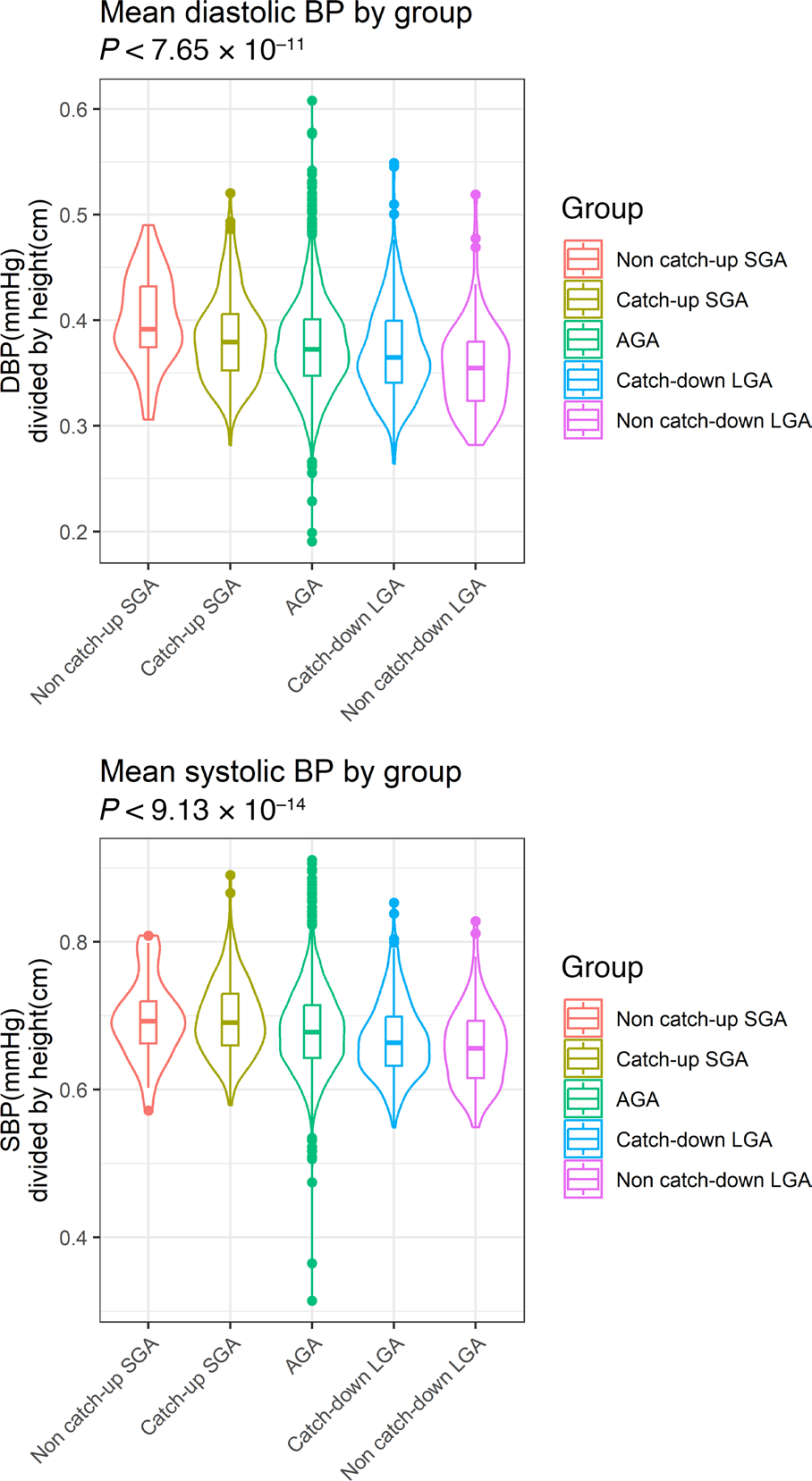
Violin plots. At age 17 years, Kruskal-Wallis tests showed that there were differences in height-corrected systolic and diastolic BPs, decreasing with group-wise increase in height.

In the ALSPAC cohort, groups based on birthweight, gestation, and CU status were generated — SGA with and without CU growth, those appropriate for gestational age (AGA), and those large for gestational age (LGA) with or without catch-down growth at expected frequencies (Figure 2). At age 17 years, there were differences in height-corrected systolic blood pressure (SBP) and diastolic BP (DBP) (Figure 3). Assessment of variation across both the transcriptome and the epigenome, using partial least squares discriminant analysis (PLSDA), demonstrated that the CU-SGA group differed from the rest of the cohort. Specifically, differentially methylated regions were identified in *PON1* and *SLC44A2*, both associated with lipid metabolism (*P* < 6.7 × 10^–5^).

**Figure 4:**
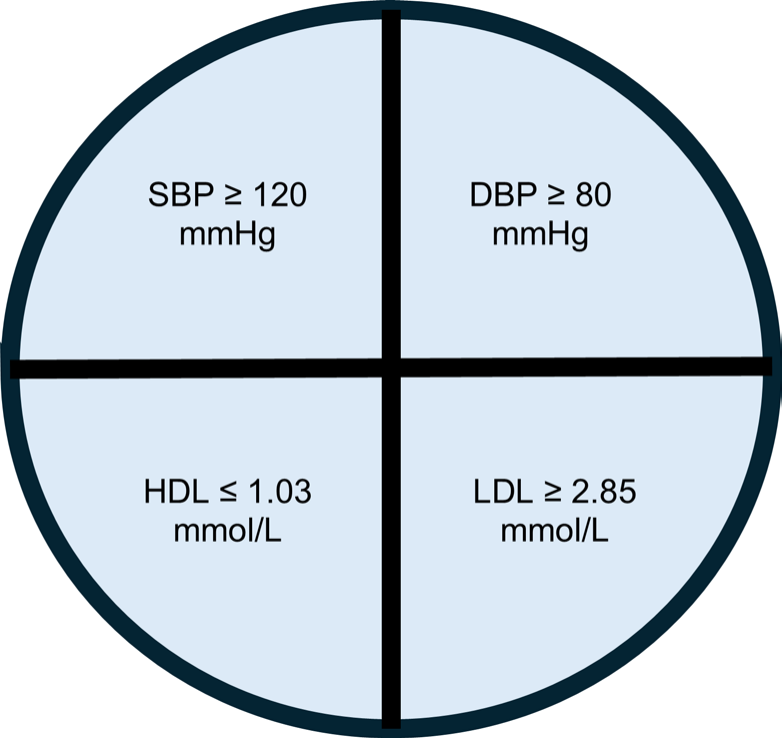
NHLBI criteria. If any one of these criteria were present, then the participant was classified as having an unhealthy/prehypertensive phenotype.

**Figure 5:**
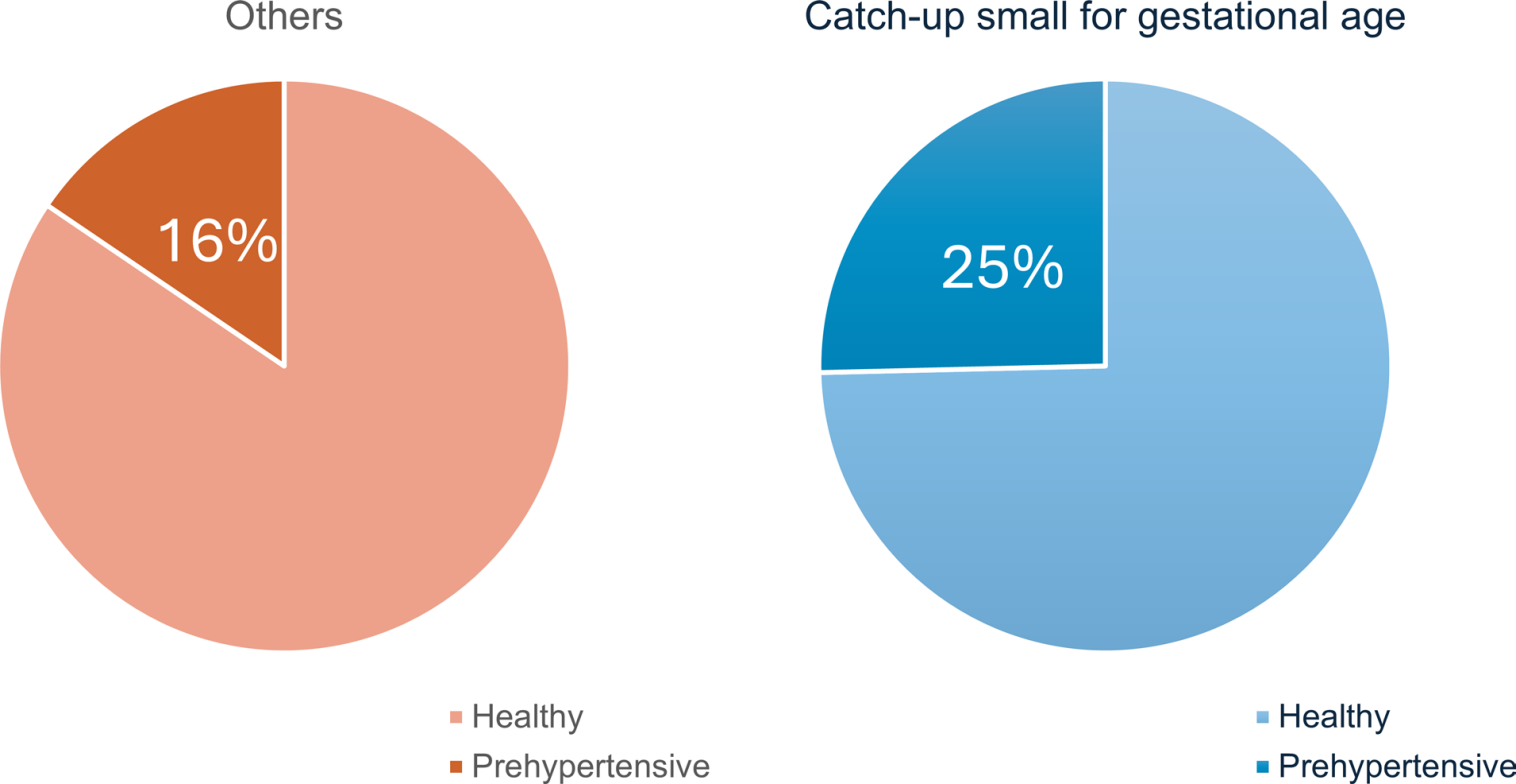
Comparisons between individuals with catch-up SGA (CU-SGA) and others. At age 17 years, individuals with CU-SGA were 1.6 times more likely to be in the prehypertensive group than others; 155 of 611 (25%) of the individuals with CU-SGA had developed a prehypertensive phenotype.

To further investigate genomic differences between the CU-SGA group and the rest of the cohort and to determine whether there was a relationship to cardiovascular risk, the 2 groups were subclassified on the National Heart, Lung and Blood Institute (NHLBI) criteria for the presence of prehypertension at age 17 years (Figure 4). Based on this NHLBI risk score, 2,134 individuals at risk of prehypertension were identified. Individuals with CU-SGA were 1.6 times more likely to be in the prehypertensive group than other children (*P* < 1 × 10^–5^); 155 of 611 (25%) of the individuals with CU-SGA had developed a prehypertensive phenotype (Figure 5). The prehypertensive CU-SGA group also had a higher height velocity than healthy individuals with CU-SGA, during their CU phase (*P* = 0.03). Overall, these data indicate that it is only a subset of the CU-SGA group that have a raised cardiovascular risk profile at age 17 years — hereafter referred to as “unhealthy CU-SGA.” Further analysis was undertaken on this group compared with the rest of the cohort.

**Figure 6:**
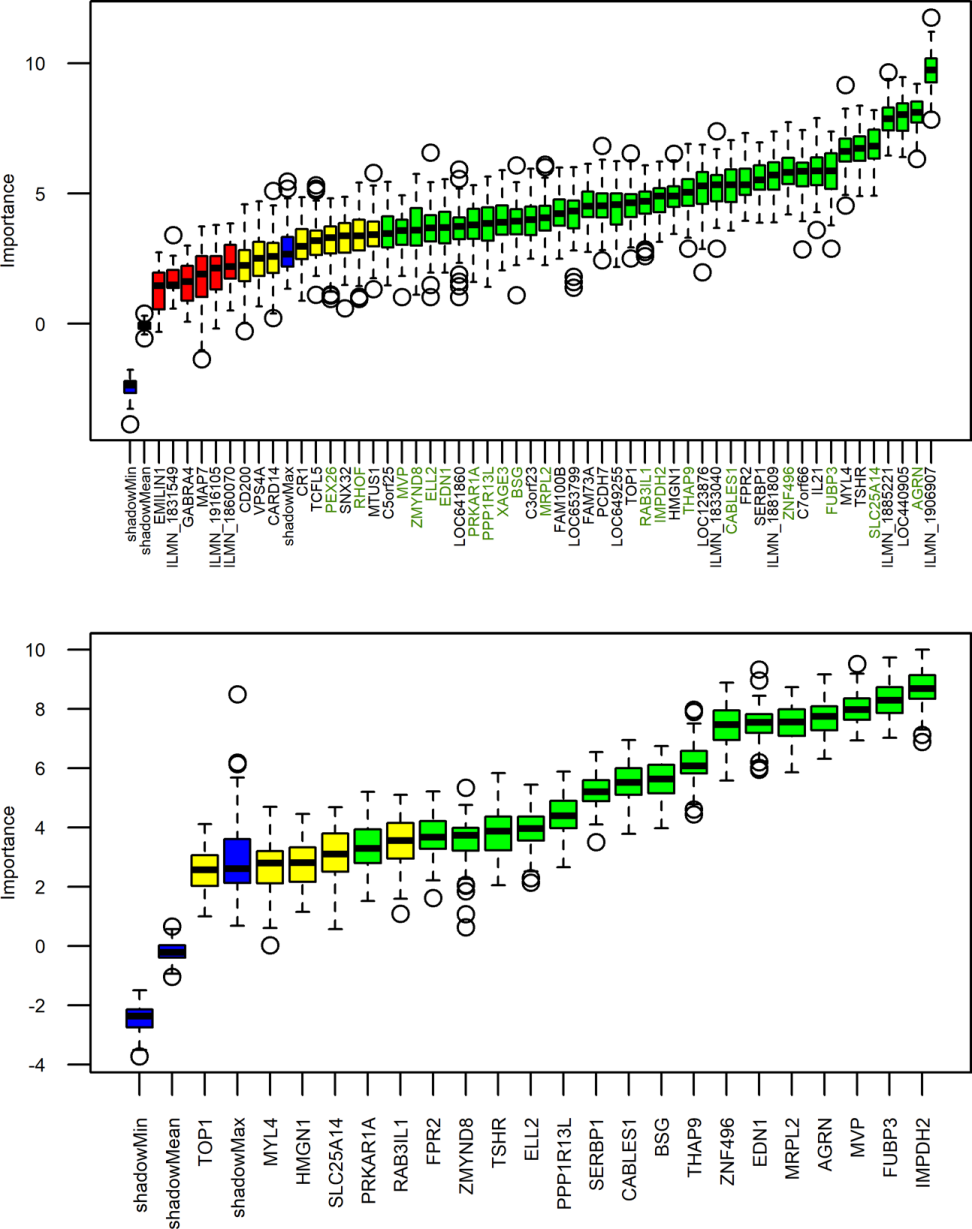
Boruta plots. All genes confirmed as informative are shown in green, those that were deemed tentative in yellow, and those uninformative in red. Random forest based on genes refined from a hypergraph found that 42 genes were predictive of prehypertensive CU-SGA in the ALSPAC cohort (left). Thirty-three of the 42 were present in the BabyGRO transcriptomic dataset, of which 20 were predictive of the upper quartile of childhood SBP in the BabyGRO cohort (OOB AUC 0.971, OOB error rate of 3.6%). These 20 genes are shown in green on the right hand side figure and also indicated by arrows on the left Boruta plot.

Differentially methylated points (DMPs) in the epigenome and differentially expressed genes (DEGs) in the transcriptome, which were correlated, were used in a hypergraph approach to define higher-order interactions. This resulted in the identification of a subset of 54 genes that distinguished the unhealthy CU-SGA group from the rest. Using random forest (RF), 42 of these 54 genes were informative in separating the groups (AUC 0.984, out of bag [OOB] error rate 5.4%; Figure 6). By searching the GWAS catalog (15), associations between these 42 genes and height, BMI and BMI-adjusted hip circumference, high-density lipoprotein (HDL) cholesterol, low-density lipoprotein (LDL) cholesterol, fatty acid measurements, and circulatory cells — e.g., platelet count and urea/uric acid cycle metabolites— were identified. This demonstrated that unhealthy individuals with CU-SGA can be distinguished from all other young adults in this cohort using transcriptome and epigenome data from earlier childhood.

Identifying these genes from an at-risk subgroup within a normal population raised the question of whether the same set of genes could predict an adverse cardiovascular profile in an independent cohort at a younger age. To test this, data from the Manchester BabyGRO cohort were used. This cohort was enriched for children in early-life (aged 3–7 years) who had been born following pregnancies with SFG (74%), where only 11% were SGA at birth.

**Figure 7:**
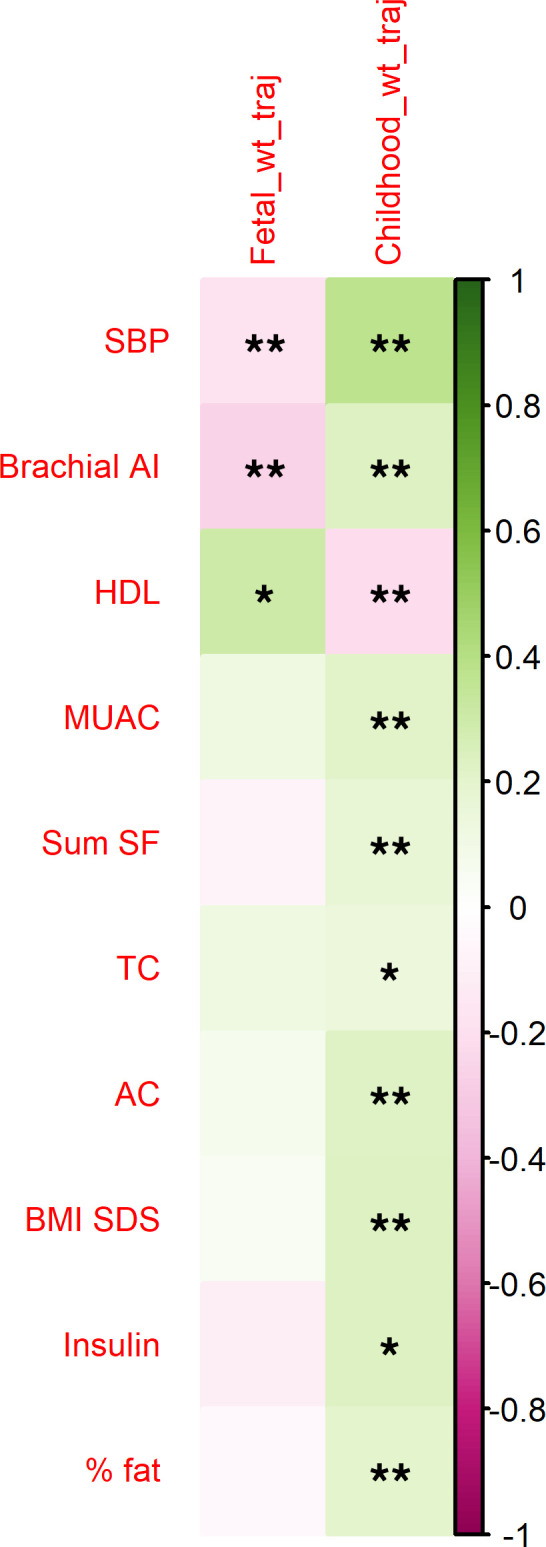
Relationships between fetal and childhood weight trajectories and childhood markers of cardiometabolic risk in children from the Manchester BabyGRO Study. Spearman’s rank tests indicated that fetal weight trajectory correlated with systolic blood pressure (SBP) and brachial augmentation index. A trend toward significance was observed for high-density lipoprotein (HDL). Childhood weight trajectory correlated with numerous markers of cardiometabolic health in a cohort children enriched for pregnancies with suboptimal fetal growth but where only a minority were small for gestational age-born.

In order to verify that this cohort was a relevant test set, correlations were established between fetal and childhood weight trajectories and childhood markers of cardiometabolic risk (Figure 7). A triad of correlations between change in fetal weight (Δfetal wt), change in child weight (Δchild wt), and SBP was demonstrated. Of the childhood cardiometabolic risk markers, SBP displayed the strongest association with Δchild wt (tau = 0.37, *P* = 2.896 × 10^–6^), and this remained when controlling for height SDS (tau = 0.37, *P* = 2.427 × 10^–6^). Differences between quartile 1 (Q1; lowest intrauterine percentile change) and Q4 (highest intrauterine percentile change) of Δfetal wt were found for HDL (lower in Q1) and SBP (higher in Q1) (both *P* < 0.05; Table 2). Furthermore, comparisons between Q1 (lowest childhood weight SDS change) and Q4 (highest childhood weight SDS change) of Δchild wt revealed differences in SBP, BMI SDS, abdominal circumference (AC), mid-upper arm circumference (MUAC), brachial augmentation index (AI), and fat mass percentage (%fat, all lower in Q1; *P* < 0.05), with differences in HDL (higher in Q1; *P* = 0.06) and insulin (lower in Q1; *P* = 0.09) trending toward significance (Table 3). The transcriptome in participants from the BabyGRO study was assessed by k means clustering, which clusters groups without any a-priori assumptions. Two clusters were identified, which differed in SBP (median 111 mmHg [range 101–134], *n* = 7 versus 102 [range 89–122], *n* = 24, *P* = 0.009) but did not differ in any other markers of cardiometabolic risk, birthweight, Δfetal wt, or Δchild wt. This suggested that prediction of SBP using the genes derived from ALSPAC in the BabyGRO cohort would be appropriate.

Using RF based on the genes predictive of prehypertensive CU-SGA in the ALSPAC cohort, the upper quartile (Q4) of childhood SBP in the BabyGRO cohort could be predicted with an OOB AUC of 0.971 and an OOB error rate of 3.6%.

## Discussion

Our study demonstrated that individuals who were CU-SGA were more likely to have prehypertensive phenotypes. Within ALSPAC, 155 of 611 (25%) of CU-SGA children had developed an unhealthy phenotype by the age of 17 years. This prehypertensive CU-SGA group had a higher height velocity than healthy individuals with CU-SGA, during their CU phase (*P* = 0.027), potentially adding support for a relationship between growth trajectories and cardiovascular risk.

To understand the relevance of this greater height velocity, we assessed the heights of children in the BabyGRO cohort in relation to parental heights where available (*N* = 60). At the time of the clinic visit, 27% (16 of 60) had height percentiles that were greater than 1 SDS above their calculated midparental height, suggesting excessive or overshooting of CU height gain.

Having defined the subset of prehypertensive CU-SGA, we wanted to explore the underlying genomic landscape and distill this down to the most functionally connected expressed genes. A hypernetwork approach led to refinement of 42 genes relating to anthropometry and markers of glucose metabolism, lipid metabolism, and vascular health. Then, a further machine learning approach, RF, enabled us to determine the predictive power of these genes, demonstrating that an unhealthy phenotype at age 17 years can be predicted from childhood ‘omics.

A total of 75% of CU-SGA children did not develop unhealthy phenotypes by 17 years. This raised the question about why some children develop adverse cardiometabolic phenotypes, while others do not. To understand this, we assessed the NHLBI criteria that placed CU-SGA in the unhealthy group, and this was high SBP first, low HDL second, high LDL third, and DBP fourth, which indicates that high SBP was the primary driver. In addition, we found that when CU-SGA and LGA-catch down are grouped together and compared against the rest of the cohort, they had higher BMI SDS, suggesting that those with the greatest deviation in their growth trajectory in early childhood are more likely to be in the unhealthy group. Existing literature supports a relationship between early life adiposity gain and later life cardiometabolic disease (16). A GWAS has demonstrated that fetal genetic factors play a major role in governing both the low birthweight and greater later-life risk, involving insulin signaling, glucose homeostasis, chromatin remodeling, and glycogen biosynthesis (17). In line with these findings, we have demonstrated that a set of genes that is detectable in childhood can predict prehypertension at age 17 years, and the same set of genes can predict higher SBP at age 3–7 years, suggesting that searching for this specific ‘omic signature in infancy could indicate which individuals are predisposed to cardiometabolic risk factor development.

The cumulative effect of 42 genes resulted in their strong predictive ability within a RF model (Figure 6). Our RFs generated OOB AUCs of 0.984 and 0.971 to predict the prehypertensive group in ALSPAC and the upper quartile of SBP in BabyGRO, respectively. Previous studies in patients with malignancy (18, 19) and cardiovascular morbidity (20) report AUCs of ≥ 0.8, considering these highly predictive. Moreover, other studies that consider their RF classification algorithms as clinically applicable report much higher error rates (25%–31%) (21, 22) than those generated by our models (5.4% [ALSPAC model] and 3.6% [BabyGRO]). These studies also lack an external validation dataset, using data from within the same cohort to provide internal validation alone. This highlights a particular strength of our work.

The earliest age at which ‘omic samples were collected in ALSPAC was 7 years. To move the focus onto early life and understand which risk markers could be identified in early childhood, we recruited a cohort of younger children, with the majority experiencing SFG, in which we identified differences in HDL and SBP between quartiles of both fetal and childhood weight trajectory (Table 2 and Table 3). This supported the use of this cohort as a validation set, to test the genes identified from the ALSPAC cohort. A separate approach where only the number of clusters was predefined (but otherwise unsupervised), k means clustering, identified 2 groups which differed in SBP but no other cardiometabolic markers, suggesting that prediction of SBP should be evaluated.

Having defined the genes to test from ALSPAC, justifying the suitability of the BabyGRO cohort as a validation set and establishing SBP as the risk marker to predict, we demonstrated that ALSPAC genes predict the upper quartile of SBP in BabyGRO. This implies that genes associated with an adverse early-life growth trajectory that predict late adolescent prehypertension can also predict SBP in early childhood.

ALSPAC was a population-based study, where participants were selected from a specific geographical area and time period. A number of other cohorts have been formed in the same way (3, 23–25), allowing for recruitment of large numbers and sufficient power to identify at-risk subgroups. Examples of such studies are the Southampton Women’s study (*N* = 1,216) (3, 26) and the Born in Bradford study (*N* = 13,766) (24), where key ethnic differences in fetal growth trajectories have been demonstrated (27). Other cohorts of normal healthy pregnancies have been used to investigate relationships between infant weight gain and cardiometabolic risk development in children (28), and it is well established that CU or accelerated growth plays a major role in the development of long-term cardiometabolic morbidity (29).

Despite this, plus clear evidence that there is a strong genetic component to postnatal growth (30), there are few large-cohort studies with availability of ‘omic markers in childhood. The Generation R cohort (*N* = 9,778) (23) recruited pregnant women with delivery dates between 2002 and 2006, with cord and peripheral blood DNA methylation samples collected at ages 6 and 10 years. Important negative findings relating to epigenetic aging and a lack of association with BP, carotid intima media thickness, and carotid distensibility in school-aged children have been reported (31). In Born in Bradford, large metabolomic datasets (32) are available with NMR data from 7,980 and LCMS data from 1,000 cord blood sample, which could provide further insights. The more recent Singapore Preconception Study of Long-term Maternal and Child Outcomes (S-PRESTO) study, which was set up to examine determinants of maternal and child future health, includes genetic, and epigenetic (longitudinal DNA methylation profiling) samples (33). While these datasets are available and a need for disease prevention strategies is recognized (34), they have not been utilized in the context of cardiovascular risk prediction. In our study, through utilizing a hypergraph approach, refining sets of genes that are not simply correlated, but coordinated across higher orders (35), we have demonstrated for the first time that combining multi-omic data with fetal and child growth trajectories can lead to refinement of a set of predictive genes. This is a potentially powerful approach for assessing and understanding DOHaD that could be applied in current and future studies.

Studying GWAS associations of individual genes allowed us to identify groups of genes that may be related and may indicate important pathways (Supplemental Tables 1 and 2; supplemental material available online with this article; https://doi.org/10.1172/jci.insight.192837DS1). *RHOF* was ranked the most important gene in the prediction of higher SBP in BabyGRO. The Ras homolog (Rho) family of GTPases is a family of G proteins, known to be involved in cell contractility and assembly of the cytoskeleton. Rho protein signaling has been extensively studied in relation to the development of hypertension (36). Nitric oxide–mediated signaling in vascular smooth muscle cells, essential for the regulation of vascular tone and arterial resistance, is a target and effector of RhoA and RAS1 signaling. Activation of RhoA and Rho kinase decreases endothelial nitric oxide synthase (eNOS) production through numerous mechanisms. As examples, RhoA-Rho kinase signaling negatively regulates eNOS function by inhibiting the PI3/AKT signaling pathway or through stimulation of arginase activity.

Within our 20 genes, a number of GWAS associations was present with components of the urea cycle (urea, uric acid, citrulline). These were *FUBP3*, *SERBP1*, and *THAP9*. This may be of relevance, since arginase is the final enzyme of the urea cycle, catalyzing the conversion of arginine (a precursor for nitric oxide) to ornithine and urea.

In relation to body height*, CABLES1* and a further 5 genes were identified: *AGRN*, *FUBP3*, *FPR2*, *PEX26*, and *PRKAR1A*. In relation to lipid metabolism, FPR2 is a G protein coupled receptor that is expressed in vascular smooth muscle cells. It is activated by serum amyloid A (37, 38). Accumulation of serum amyloid A (SAA) leads to loss of the protective association between HDL and cardiovascular risk (39, 40). Additionally, the presence of *TSHR* (although deemed tentative, despite its higher position in the hierarchy) within the 21 identified may suggest a role for thyroid hormones in the development of a metabolically unhealthy phenotype. Hypothyroidism is associated with increased serum cholesterol, LDL, and triglycerides due to the modulatory effect of thyroid hormones on cholesterol production, transformation and clearance (41). Lastly, in relation to glucose metabolism, *PRKAR1A* plays an important role in insulin secretion, with evidence of insulin resistance demonstrated in the KO mouse (42) and also in humans with inactivating mutations (Carney complex) (43).

### Limitations.

While the presence of a validation set and measures across multiple time points was a particular strength of our work, ‘omic data prior to early childhood were not available. Also, the relevance of these predictive markers could be explored at older ages.

CU growth was defined on the basis of height at 7 years in ALSPAC, whereas associations with childhood weight trajectory were determined in BabyGRO, with paucity of birth length data to examine CU defined by height. However, when we recategorized the birth size groups in ALSPAC using weight instead, the groupings remained largely the same.

Small numbers within BabyGRO may also be considered a limitation of the study. However, the study design using a large population-based study to define an ‘omic signature, with the BabyGRO study including deep phenotyping of participants within a cohort enriched for SFG allowed us to focus on patterns of fetal weight change instead of birthweight alone.

The ALSPAC cohort participants were predominantly a white (only 4.1% non-white mothers compared to 7.6% in the whole of Great Britain in 1991), affluent (68.7% owner occupied accommodation compared to 63.4% in the whole of Great Britain) population (44). Previous studies in this field have commented on the challenges of generalizability from findings (45). While this should be considered, the validation cohort represented a more ethnically diverse population (68% White, 16% Asian, 12% Black, 4% other), and further validation in more ethnically diverse populations is required.

### Conclusion.

An unhealthy CU-SGA group has been defined, and resources could be focused on minimizing future risk for this subgroup. A transcriptomic signature has been refined, which can predict both prehypertensive phenotype in adolescence in a healthy normal cohort and the upper quartile of SBP in early childhood. This signature needs to be tested, validated, and characterized in other cohorts, including during infancy, before its potential clinical use as a test for early identification of individuals at greatest risk of cardiometabolic disease.

## Methods

### Sex as a biological variable.

Both males and females were included within both cohorts.

### The ALSPAC cohort.

The ALSPAC is a prospective observational study that involved recruitment of 14,541 pregnancies (14,676 fetuses) over 1991 and 1992, from a specific geographical region in the Southwest of England (Figure 1). Data are available to study genetic, biological, environmental, and psychosocial factors affecting health (4, 46). From maternal data available (*N* = 10,000), 0.4% were reported to have existing diabetes, 0.5% gestational diabetes (47), 2% preeclampsia, and 14.5% had gestational hypertension (BP above 140/90 without proteinuria) (48). There were 14,062 live birth,s and 13,988 children were alive at 1 year. Since the incidence of SGA (< tenth percentile) in those with gestational hypertension is 15% (49), only 2.3% of our cohort were estimated to have gestational hypertension and SGA. Therefore, the decision was made to include the entire cohort in the analysis. In total, 12,447 of these were term deliveries (above 37 weeks’ gestation). Their growth has been documented from birth, through childhood and adolescence into adulthood. A wide range of anthropometric parameters as well as BP and blood profiles have been collected throughout the study. In this study, the focus has been on birth characteristics, childhood growth and cardiovascular markers in late adolescence. When the oldest child was aged 7 years, further recruitment was undertaken to boost the sample size. The total was therefore 15,454 pregnancies and 15,589 fetuses, of which 14,901 were alive at 1 year (Figure 1). Epigenomic (Illumina Infinium 450K) and transcriptomic (Illumina Beadchip) data were collected from 980 children aged 7 and 947 children aged 9 years, respectively (Figure 1); this provided an adequately sized dataset to undertake analyses on the relationship of omic profiles to cardiovascular status in groups defined by growth trajectories.

Children were allocated to groups based on their birthweight and gestation. Those below the tenth percentile were classified as SGA and those above the 90^th^ were classified as LGA. CU and catch-down (CD) status by the age of 7 years was also defined: those who had returned to above the tenth or below the ninetieth percentile in height were deemed to have caught up or down, respectively. The largest group were those born AGA. Differences in late adolescent phenotype (growth and cardiovascular status at 17 years) between these groups were assessed using mixed effect models normalized for height and BMI and corrected for false discovery rate, with *P* < 0.05 considered significant. Cardiovascular status at 17 years was defined by SBP, DBP, and lipid profiles.

Further characterization of cardiovascular status was undertaken on the whole cohort focusing on CU-SGA group versus the rest of the cohort using the NHLBI criteria for prehypertension (Figure 1 and Figure 5). This identified those in the CU-SGA group and the rest of the cohort with a prehypertensive phenotype.

### Transcriptomic analyses in ALSPAC.

Using the epigenome data available at age 7 years (*N* = 980) and the transcriptomic data at age 9 years (*N* = 947) on the CU-SGA group and the rest of the cohort, an initial assessment of the underlying differences was made using PLSDA, a dimensionality reduction tool, to potentially detect overall differences between groups and thereby inform the decision on whether to perform further analyses. As PLSDA revealed differences between CU-SGA (CU-SGA) and the rest of the cohort, further omic analyses were undertaken using the CU-SGA group with a prehypertensive phenotype versus the rest of the cohort.

Using a supervised approach, DMPs and DEGs between the prehypertensive CU-SGA group and all other groups were determined (50, 51).

**Figure 8:**
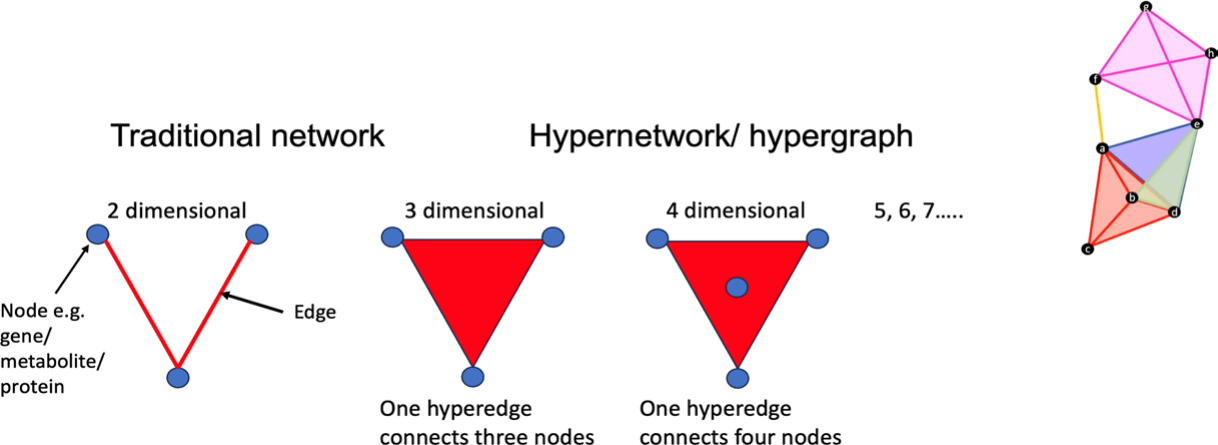
Hypergraphs. In a traditional network, an edge (represent here by the line) connects 2 nodes together. In a hypergraph, an edge can represent a relationship between any number of nodes — e.g., multiple proteins interacting to form a protein complex. Not only can edges connect any number of nodes, but the same pair of nodes can be connected by multiple edges. Altogether, this allows us to increase the depth of the information we can capture. The hypergraph central clusters represent subsets of significant genes and metabolites sharing the most correlations and indicating likely functional relationships.

DMPs and DEGs were integrated to identify functional links between 520 CpG islands (CpGs) at age 7 years and 55 DEGs at age 9 years. This approach considers genomic regions with 7 or more DMPs to be differentially methylated regions. The identified CpGs were then grouped into chromosomal regions to identify genes with known associations with lipid metabolism. The DMPs and DEGs, which were correlated, were used as the gene set for a hypergraph approach to determine higher order interactions between these genes (Figure 8).

The hypergraph enabled the identification of a set of 54 DEGs: these were tested for their predictive value, using a RF model of 1000 trees (Figure 1, Step 5). The R package, ‘Rattle’ (52) was used. For each model, a confusion matrix was produced, allowing the generation of a receiver operating characteristic (ROC) curve (based on true positive and false positive rates), with an associated AUC and an error rate. As RF randomly selects samples in each iteration to derive patterns which distinguish each group (known as training), some samples are left unselected in each iteration and can be used to test the predictive capacity of the model during training. These are known as OOB metrics and are particularly useful for the analysis of datasets where there are insufficient samples to generate independent training and testing datasets. Boruta (53) plots illustrating the ranking of the variables of importance (VIPs) were also generated, identifying genes as important, tentative, or rejected in the RF model.

### The BabyGRO cohort.

An independent test cohort from early childhood was recruited (Figure 1), to address whether the genes refined from the hypergraph could be used to predict cardiovascular status at this age. This cohort included offspring of pregnancies demonstrating a range of fetal and childhood growth trajectories (Figure 1 and Table 1).

**Figure 9:**
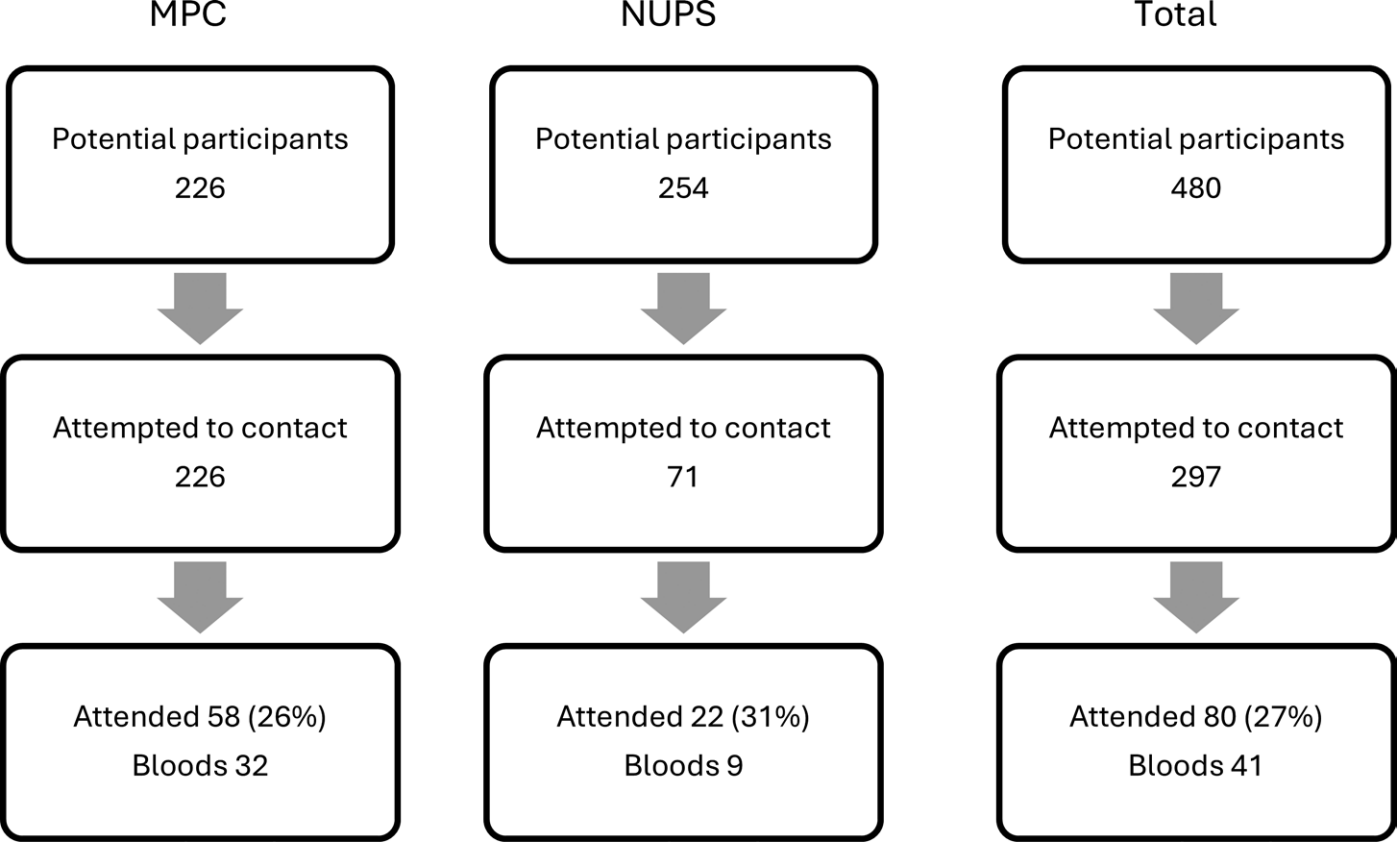
The BabyGRO cohort. In total, 226 eligible mothers who had been seen in the Manchester Placenta Clinic due to abnormal serology antenatally, where both mother and child were alive and the child was aged > 3.0 and < 7.0 years, were approached for recruitment. Targeted recruitment of children born following pregnancies in the New Ultrasound Parameters Study involved approaching those aged 5.7 years (the median age of Manchester Placenta clinic recruits) ± 1 year, whose birthweight was > tenth and < ninetieth percentiles. Following this process, a recruitment pool of 71 was established. In total, 80 children were seen and 41 also had a fasting blood test. NUPS, New Ultrasound Parameters Study.

Children aged 3–7 years were recruited from 2 sources. The first was a group of 226 children whose mothers had been seen in the Manchester Placenta Clinic (MPC), a tertiary service offering obstetric and midwifery care to women following detection of greater FGR risk based on abnormal maternal antenatal serology (pregnancy associated plasma protein-A < 0.415 multiples of the median [MoM], α fetoprotein >2.2 MoM or inhibin A >2 MoM), where the pregnancies resulted in livebirths above 34 weeks’ gestation and who had given consent to contact for future research. The second source comprised 71 offspring of 254 women who had taken part in a study involving uncomplicated pregnancies, resulting in livebirths above 34 weeks’ gestation with birthweights between tenth and ninetieth percentiles. Pregnant women with either preexisting or gestational diabetes were excluded. This cohort had a median age of 5.7 years ± 1 year, to match the median age of participants from the MPC (22). In total, 58 and 22 children were recruited from each pool respectively (Figure 9). Exclusion criteria were lack of understanding (despite an interpreter), maternal age < 16 years, congenital anomaly or presence of a medical condition affecting growth or cardiometabolic risk.

Height (to the nearest 0.1 cm) was measured using a Harpenden stadiometer. Weight (to the nearest 0.1 kg) was measured and BMI calculated as weight (kg) divided by height (m^2^). MUAC, AC and thigh circumference (all to the nearest 0.1 cm) were measured. Biceps, triceps, subscapular, suprailiac, and abdominal skinfold thicknesses (nearest 0.2 mm) were measured using Holtain calipers, and the sum calculated. Body composition was measured by air displacement plethysmography (BODPOD, Cosmed) (54). This follows a similar principle to hydrostatic weighing, the gold standard for body composition assessment. The %fat, fat free mass (grams) and fat free mass percentage (%) were recorded.

The Tensiomed Arteriograph (Tensiomed) was used to quantify brachial AI, a measure of the strength of wave reflection, calculated as the ratio of the amplitude of the reflected wave), with SBP and DBP also recorded.

As serological markers of cardiometabolic risk, the children had fasted peripheral blood samples collected for glucose, insulin, cholesterol, HDL, LDL, triglycerides, IGF-I, cholesterol, total cholesterol/HDL ratio and non-HDL cholesterol measurements using clinical-grade assays.

For transcriptomics, 2.5 mL of blood was collected into a PAXgene tube. RNA extractions were carried out according to manufacturer instructions and RNA stability was maintained by storage at –80°C prior to analysis at the Core Facility. For RNA-Seq, quality and integrity of the RNA samples were assessed using a 4200 TapeStation (Agilent Technologies) and then libraries generated using the TruSeq Stranded mRNA assay (Illumina, Inc.).

### Transcriptomic analyses in BabyGRO.

The R package, edgeR (51) was used to process transcriptomic data and produce counts per million (cpm, a measure for the expression level of a gene) values. Principal component analysis plots and histograms of cpm values were compared and demonstrated similar distributions of scaled and unscaled data and illustrated that no further processing was necessary.

Unsupervised k-means clustering separated participants into 2 groups (Figure 1). Differences in phenotype were examined using *t* tests and Mann-Whitney *U* tests. This provided evidence to support the evaluation of SBP as a cardiometabolic marker to use in a RF classification model, to test the predictive ability of the ALSPAC hypergraph-derived genes. This set of 42 genes from the ALSPAC cohort were compared with genes within the BabyGRO study: transcriptomic data on 33 of these genes were available. These were then used in a RF model with 1,000 trees to test the ability of the ALSPAC-derived genes to predict the upper quartile of SBP in the BabyGRO cohort (Figure 1).

### Statistics.

The Δfetal wt was calculated as birthweight percentile minus 23-week estimated fetal weight percentile (as calculated by the Hadlock formula and percentiles calculated, divided by the number of days in between) (55, 56). The Δchild wt was calculated as weight SDS minus birthweight SDS, divided by years (Figure 1). Quartile cut-offs were determined within the cohort. Two-tailed Student’s *t* tests (parametric) and Mann-Whitney *U* tests (nonparametric) were used to establish differences in cardiometabolic markers between quartiles of weight trajectories (Table 2 and Table 3).

### Study approval.

The Manchester BabyGRO study was approved by the Northwest Research Ethics Committee (Manchester, United Kingdom, REC reference 17/NW/0153, IRAS ID 187679) and the UK Health Research Authority. Written informed consent was obtained from all participants in accordance with the Declaration of Helsinki.

### Data availability.

Values for all data points in graphs are reported in the Supporting Data Values file. Further data that support the findings of this study are available from the corresponding author upon reasonable request.

## Author contributions

RP, TG, and PEC made equal and substantial contributions to the conception of the work, its acquisition, analysis and interpretation, drafting, and critical review of the manuscript. PGM contributed to the conception of the work, data acquisition from the ALSPAC team, and critical review of the manuscript. AR contributed to data analysis and critical review. LEH, EDJ, and AS contributed to the conception of the work; its acquisition, analysis, and interpretation; drafting; and critical review. All authors give final approval of the version to be published.

## Funding support

This work is the result of NIH funding, in whole or in part, and is subject to the NIH Public Access Policy. Through acceptance of this federal funding, the NIH has been given a right to make the work publicly available in PubMed Central.

- Centre Grant to the Maternal and Fetal Health Research Centre by Tommy’s The Pregnancy and Baby Charity
- Child Growth Foundation
- European Research Council funding as part of the Health and Environment-wide Associations based on Large Population Surveys (HEALS) study

## Supplementary Material

Supplemental data

ICMJE disclosure forms

Supporting data values

## Figures and Tables

**Table 3:**
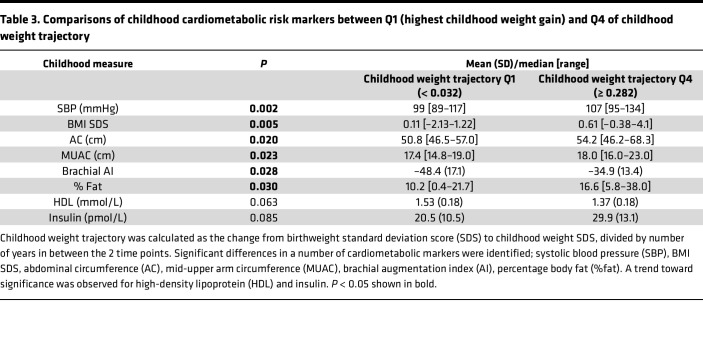
Comparisons of childhood cardiometabolic risk markers between Q1 (highest childhood weight gain) and Q4 of childhood weight trajectory

**Table 1:**
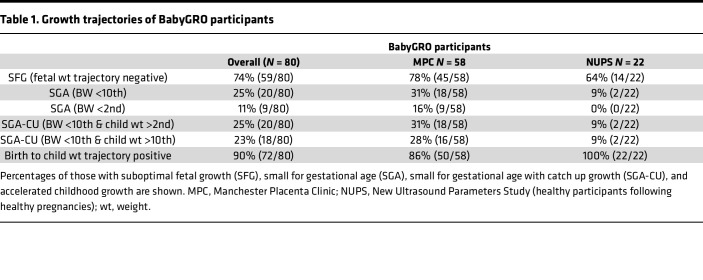
Growth trajectories of BabyGRO participants

**Table 2:**
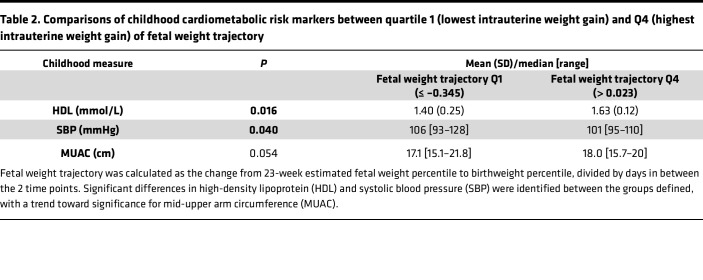
Comparisons of childhood cardiometabolic risk markers between quartile 1 (lowest intrauterine weight gain) and Q4 (highest intrauterine weight gain) of fetal weight trajectory

## Version 1 12/08/2025

Electronic publication
