# Supplementary material for: A transcriptomic signature that predicts prehypertension in adolescence and higher systolic blood pressure in childhood
Source: JCI Insight. 2025 Dec 8;10(23):e192837. doi: 10.1172/jci.insight.192837 (PMC12890495; doi:10.1172/jci.insight.192837)
Supplement: Supplemental data [file jciinsight-10-192837-s168.pdf]

Supplemental Table 1. Genes predictive of higher systolic blood pressure in BabyGRO. 20 genes that predicted a prehypertensive phenotype in ALSPAC were also predictive of the upper quartile of systolic blood pressure in children aged three to seven years.

Gene	Phenotypes from GWAS catalog
AGRN	blood protein measurement, educational attainment, blood phosphate measurement, body height, brain measurement
SLC25A14	educational attainment
ZNF496	platelet volume, platelet distribution width, reticulocyte volume, neutrophil count
FUBP3	body height, heel BMD, citrulline measurement, body weights and measures/body height, femoral neck BMD
SERBP1	IBS, Crohn's, ulcerative colitis, primary biliary cirrhosis, serum urea measurement
FPR2	blood protein measurement, alcohol drinking, HDL cholesterol measurement, alcohol consumption & HDL, body height
CABLES1	body height, platelet count, mean platelet volume, body weights and measures/body height, BMI-adjusted waist circumference
THAP9	monocyte percentage of leukocytes, leukocyte count, blood protein measurement, granulocyte percentage of myeloid white cells, urate measurement, bone density
IMPDH2	reticulocyte count, blood protein measurement, IBD, eosinophil count, reticulocyte measurement
RAB3IL1	omega-3 polyunsaturated fatty acid measurement, fatty acid measurement, linolenic acid measurement, lysophosphatidylethanolamine measurement, lysophosphatidylcholiine measurement, phosphatidylethanolamine measurement
MRPL2	haemoglobin measurement, diffuse plaque measurement
BSG	mean reticulocyte volume, educational attainment, endometriosis, late onset Alzheimer's disease
PPP1R13L	Alzheimer's, HDL cholesterol measurement, CRP measurement, heel BMD, haematocrit, erythrocyte count
ELL2	reticulocyte measurement, reticulocyte count, aortic measurement, total blood protein measurement, serum non albumin protein measurement
ZMYND8	platelet volume, BMI adjusted waist-hip ratio, heel BMD, waist-hip ratio, BMI adjusted hip circumference
PRKAR1A	alkaline phosphatase measurement, body height, reticulocyte count, reticulocyte measurement, glucose measurement
EDN1	vasoactive peptide measurement, BMI, haematocrit, erythrocyte count haemoglobin measurement
MVP	RBC density measurement, MCV, MCH, erythrocyte count, MCH
RHOF	platelet volume distribution width, count
PEX26	non-alcoholic fatty liver disease, alkaline phosphatase measurement, body height, cortical thickness, COPD

Supplemental table 2. Additional genes predictive of a prehypertensive phenotype at age seventeen years in ALSPAC. These genes predicted a prehypertensive phenotype in ALSPAC but were not present within the 20 genes predictive of higher systolic blood pressure in BabyGRO.

Gene	Phenotypes from GWAS catalog
ILMN_1906907	-
LOC440905	pseudogene therefore no GWAS data
ILMN_1885221	-
MYL4	blood urea nitrogen measurement, platelet count, thyroid preparation use measurement, BMI-adjusted hip circumference, serum urea measurement
TSHR	thyroid stimulating hormone measurement, Grave's disease, hyperthyroidism, apolipoprotein A1 measurement, HDL cholesterol measurement
IL21	atopic eczema, mosquito bite reaction size measurement, coeliac disease, mosquito bite reaction itch intensity measurement, asthma
ILMN_1881809	-
C7orf66 ILMN_1833040	insomnia, protein measurement, self-reported comparative body size age 10, self-reported educational attainment
LOC123876	-
HMGN1	-
PCDH7	smoking initiation, brain measurement, cortical thickness, adolescent idiopathic scoliosis, ADHD
TOP1	serum GGT measurement, LDL cholesterol measurement, SHBG measurement, total cholesterol measurement, LDL cholesterol measurement, physical activity
LOC649255	**-**
FAM100B	SHBG measurement, LDL cholesterol measurement, reticulocyte measurement, BMI adjusted waist-hip ratio, triglyceride measurement
LOC653799	-
C3orf23	body height, BMI, daytime rest measurement, unipolar depression, bipolar disorder
FAM73A	GFR, FEV/FVC ratio, BMI, COPD, body height
XAGE3	prostate Ca
LOC641860	-
MTUS1	blood protein measurement, body height, FEV/FVC ratio, cup-to-disc ratio measurement, PR interval
C5orf25	RBC distribution width, body height, AFP measurement, uric acid measurement, serum albumin measurement
